# Loss of Parkinson’s susceptibility gene LRRK2 promotes carcinogen-induced lung tumorigenesis

**DOI:** 10.1038/s41598-021-81639-0

**Published:** 2021-01-22

**Authors:** Chandra Lebovitz, Nicole Wretham, Maryam Osooly, Katy Milne, Tia Dash, Shelby Thornton, Basile Tessier-Cloutier, Paalini Sathiyaseelan, Svetlana Bortnik, Nancy Erro Go, Elizabeth Halvorsen, Rachel A. Cederberg, Norman Chow, Nancy Dos Santos, Kevin L. Bennewith, Brad H. Nelson, Marcel B. Bally, Wan L. Lam, Sharon M. Gorski

**Affiliations:** 1 Canada’s Michael Smith Genome Sciences Centre, BC Cancer, 675 West 10th Avenue, Vancouver, BC V5Z 1L3 Canada; 2 Department of Molecular Biology and Biochemistry, Simon Fraser University, Burnaby, BC V5A 1S6 Canada; 3 Department of Experimental Therapeutics, BC Cancer, Vancouver, BC V5Z 1L3 Canada; 4 Deeley Research Centre, BC Cancer, Victoria, BC V8R 6V5 Canada; 5 Department of Pathology and Laboratory Medicine, University of British Columbia, Vancouver, BC V6T 1Z4 Canada; 6 Department of Integrative Oncology, BC Cancer, Vancouver, BC V5Z 1L3 Canada; 7 Department of Biochemistry and Microbiology, University of Victoria, Victoria, BC V8P 5C2 Canada; 8 Department of Medical Genetics, University of British Columbia, Vancouver, BC V6T 1Z4 Canada

**Keywords:** Cancer models, Lung cancer, Cancer genomics

## Abstract

Pathological links between neurodegenerative disease and cancer are emerging. LRRK2 overactivity contributes to Parkinson’s disease, whereas our previous analyses of public cancer patient data revealed that decreased *LRRK2* expression is associated with lung adenocarcinoma (LUAD). The clinical and functional relevance of *LRRK2* repression in LUAD is unknown. Here, we investigated associations between *LRRK2* expression and clinicopathological variables in LUAD patient data and asked whether LRRK2 knockout promotes murine lung tumorigenesis. In patients, reduced *LRRK2* was significantly associated with ongoing smoking and worse survival, as well as signatures of less differentiated LUAD, altered surfactant metabolism and immunosuppression. We identified shared transcriptional signals between *LRRK2*-low LUAD and postnatal alveolarization in mice, suggesting aberrant activation of a developmental program of alveolar growth and differentiation in these tumors. In a carcinogen-induced murine lung cancer model, multiplex IHC confirmed that LRRK2 was expressed in alveolar type II (AT2) cells, a main LUAD cell-of-origin, while its loss perturbed AT2 cell morphology. LRRK2 knockout in this model significantly increased tumor initiation and size, demonstrating that loss of LRRK2, a key Parkinson’s gene, promotes lung tumorigenesis.

## Introduction

Lung adenocarcinoma (LUAD), one of the deadliest cancers worldwide^1^, is a molecularly heterogeneous disease that originates in the distal lung, affecting smokers and non-smokers. Analyses of tumor evolution suggest that LUAD progression and patient outcome are highly influenced by subclonal heterogeneity^2^, making functional characterization of molecular alterations captured by large-scale, multi-omics efforts like The Cancer Genome Atlas (TCGA) a continued priority. In a previous analysis of TCGA LUAD RNA-seq data, we identified a marked reduction in expression of Parkinson’s susceptibility gene Leucine Rich Repeat Kinase 2 (*LRRK2*), in tumors versus matched normal lung^3^. Pathogenic links between cancer and Parkinson’s disease (PD) are emerging: while cancer cells can proliferate indefinitely, diseased neurons die prematurely, placing cancer and neurodegenerative disease at opposite ends of a spectrum of aberrant cell survival^4^. Overactive LRRK2 is a common genetic cause of PD^5^, which when inhibited or knocked out perturbs pulmonary surfactant homeostasis in alveolar type II (AT2) cells^6^—a main cell of origin in LUAD^7^. LRRK2 inhibition is poised to be the first disease-modifying therapy in PD, with a small-molecule inhibitor in early phase clinical trials^8^. While pre-clinical studies of systemic LRRK2 inhibition in primates have revealed a reversible lung phenotype, which does not affect pulmonary function tests with short-term treatment^9^, potential irreversible effects beyond respiration remain to be defined—particularly in the setting of long-term LRRK2 inhibition.

Large-scale epidemiological surveys of PD patients have revealed intriguing links between brain and lung health. PD patients, with either hereditary or idiopathic disease, are less likely to develop smoking-related cancers, with a dramatically reduced incidence rate of lung cancer (0.40) compared to the general population^10^, while ongoing cigarette smoking halves the risk of developing PD compared to neversmokers^11^. Mechanisms governing these associations have yet to be elucidated, including whether altered LRRK2 plays a mediating role. Several epidemiological studies report that LRRK2 G2019S carriers are at increased risk for various non-lung cancers^12^, while others contradict these results^13,14^; therefore, the cancer epidemiology of mutant LRRK2 remains unclear. In addition, as LRRK2 mutation carriers have been the focus of most LRRK2-related epidemiological studies of cancer incidence, an overactive LRRK2 phenotype was presupposed; consequently, to our knowledge, a role for LRRK2 *loss* in cancer pathogenesis has yet to be considered, including for LUAD.

This study explores the association of *LRRK2* transcriptional repression with established clinical, genomic and transcriptomic phenotypes assembled for TCGA LUAD patient samples, identifies a significant overlap between transcriptional changes seen in *LRRK2*-low LUAD and those documented in normal postnatal lung development in mice, and, significantly, presents the first in vivo evidence that LRRK2 loss promotes carcinogen-induced lung tumorigenesis.

## Results and discussion

### *LRRK2* reduction occurs in high-risk non-terminal respiratory unit (non-TRU) type lung adenocarcinoma (LUAD)

**Figure 1:**
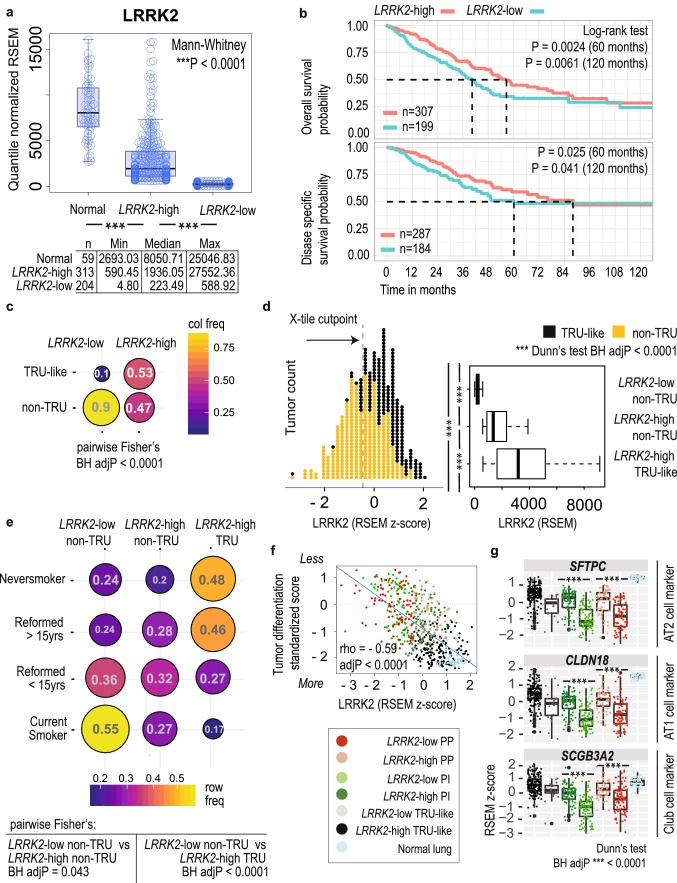
*LRRK2*-low lung adenocarcinoma is associated with poor patient survival, non-TRU expression-based molecular subtypes and worse predicted tumor differentiation. (**A**) Plot of *LRRK2* mRNA levels in LUAD tumors (dichotomized into *LRRK2*-low and -high expression groups) and adjacent normal lung tissue (RSEM values), from TCGA LUAD patients. (**B**) Kaplan–Meier plot of LUAD patient OS or DSS stratified by *LRRK2* expression status. (**C**) Pairwise Fisher’s exact test for enrichment of expression subtype frequency within *LRRK2* expression groups: TRU-like versus non-TRU type LUAD. (**D**) (Left) Standardized *LRRK2* tumoral expression per sample (RSEM values), annotated for lower risk TRU-like or higher risk non-TRU type tumors or (Right) grouped by the combined status for *LRRK2* expression and expression subtype (median centred boxplot of RSEM values). (**E**) Pairwise Fisher’s exact test for enrichment of smoking history within *LRRK2* expression groups: TRU-like versus non-TRU type LUAD. (**F**) Correlation of *LRRK2* expression with a previously published gene expression-based score representing LUAD tumor differentiation status (Spearman’s correlation coefficient − 0.59 with Holm’s adjP < 0.0001; positive scores represent increasingly poor differentiation). (**G**) Stratification of the tumoral gene expression of established markers for alveolar and bronchiolar epithelial cell types, by the combined *LRRK2* and expression subtype status of LUAD tumors (median-centred boxplot of standardized RSEM values; Dunn’s test BH adjP < 0.05).

In a previous molecular screen of autophagy-associated genes across 11 TCGA cancer types, we identified a striking reduction of *LRRK2* expression in LUAD tumors (n = 517) versus matched normal lung tissue (n = 59)^3^. To determine the potential clinical relevance of altered *LRRK2* levels in LUAD, we examined the association of *LRRK2* expression levels with clinicopathological variables. While a median split of tumor samples based on *LRRK2* expression showed a significant difference in survival between *LRRK2*-low versus -high patients (Log-rank test P = 0.033; Supplementary Fig S1), we chose to apply a supervised (and potentially more biologically meaningful) method to refine the *LRRK2* cutpoint, by deriving *LRRK2*-low and -high cohorts via optimization of a two-group cohort correlation with overall survival (OS) data, which was achieved by identifying a cutpoint that minimized the P-value defined by Kaplan–Meier OS analysis and Log-rank test (X-tile cohort separation algorithm^15^ with Monte Carlo cross-validation P < 0.05, n = 10,000 repetitions; Fig. 1A). We found that 204 of 517 (39.5%) TCGA LUAD tumors expressed a dramatic reduction of *LRRK2* expression, which stratified both overall survival (OS; Log rank test (60 months) P = 0.0024) and disease specific survival (DSS; Log rank test (60 months) P = 0.025) (Fig. 1B). Survival data was obtained from the TCGA Pan-Cancer Clinical Data Resource^16^, which was curated for high quality outcome data. Differences in sample numbers between OS and DSS analyses (Fig. 1B) occur, as only patients (from Fig. 1A) with reliable survival follow-up per outcome type were included, as determined by Liu et al.^16^ (Supplementary Table S1). *LRRK2*-low patients survived more poorly, with a hazard ratio of 1.42 (Cox proportional hazards regression adjusted for pathological stage; CI 1.06–1.91; Wald test P = 0.019; Log likelihood test P < 0.0001). Further, patients expressing the lowest amount of tumoral *LRRK2* (n = 50) had an increased OS hazard ratio of 2.42 (CI 1.19–4.90; Wald test P = 0.014; Log likelihood test P < 0.0001) and an increased DSS hazard ratio of 3.03 (CI 1.20–7.68; Wald test P = 0.019; Log likelihood test P < 0.0001), compared to patients expressing the highest amount of tumoral *LRRK2* (n = 49).

Gene expression phenotyping of multiple LUAD cohorts over the last decade^17–20^ have confirmed that non-terminal respiratory unit (non-TRU) type LUAD is associated with worse patient outcomes. To situate *LRRK2* transcriptional repression with respect to established expression-based molecular subtyping of LUAD^17,19,21^, we determined whether *LRRK2*-low tumors were enriched for higher-risk non-TRU type tumors or lower-risk terminal respiratory (TRU)-like tumors. Stratification of tumors by both *LRRK2* status and expression subtype revealed that most *LRRK2*-low tumors were of the higher-risk non-TRU type (Frequency (F) = 0.90; n = 517 patients; pairwise Fisher’s exact test, Benjamini Hochberg adjusted P (BH adjP) < 0.0001; Fig. 1C,D). Together these results associate reduced *LRRK2* expression with poor survival outcomes and the high risk non-TRU subtype of lung cancer.

### *LRRK2*-low non-TRU type tumors are associated with ongoing smoking, subtype-specific genome instability and less differentiated tumors

Non-TRU type tumors are associated with smoking, *KRAS*, *TP53* and *STK11/LKB1* mutations, and peripheral lung architecture destruction, while TRU-like tumors have established associations with never smoking, *EGFR* mutation, and preservation of peripheral lung architecture^17,22^. Non-TRU tumors can be further broken down into proximal-inflammatory (PI) tumors, enriched for *TP53* and *NF1* co-mutation, high grade solid-type morphology, and higher expression of immunotherapy targets, or proximal-proliferative (PP) tumors, enriched for *KRAS* and *STK11*/*LKB1* co-alteration and low immune-related gene expression^17,23,24^. To identify disease covariates associated with a change in *LRRK2* status, potentially independent of expression subtype, we performed an enrichment analysis for various clinical and molecular covariates in *LRRK2*-low (n = 183) versus -high (n = 146) non-TRU type tumors only.

Similar to non-TRU type LUAD in general, *LRRK2-*low non-TRU type tumors were associated with smoking history; however, patients with *LRRK2*-low tumors represented 55.5% of all ‘Current Smokers’ (pairwise Fisher’s exact test, *LRRK2*-low versus -high non-TRU BH adjP = 0.043; Fig. 1E; Supplementary Table S1), suggesting that marked *LRRK2* reduction was associated with ongoing smoking. Pathological mechanisms triggered by cigarette smoking include genome instability^25^, global gene expression changes^26^, alteration of epithelial differentiation pathways^26^, and small airway tissue remodeling^27^. At the molecular level, reduced *LRRK2* was associated with subtype-specific genome instability: *LRRK2*-low PP tumors carried higher genome-wide somatic copy number load than *LRRK2*-high PP tumors (pairwise Dunn’s test BH adjP = 0.042), while *LRRK2*-low PI tumors were more likely to have highly amplified genes (n = 77) from chromosome bands 5p12, 5p13 or 5p15 (pairwise Fisher’s exact test BH adjusted P < 0.05) (Supplementary Fig. S2 and Supplementary Tables S2, Suppl Table S3). Interestingly, somatic mutation prevalence in genes reported to be significantly mutated in LUAD^21^ (n = 52 genes) remained equivalent between tumor groups (Supplementary Tables S2), suggesting *LRRK2* transcriptional repression was not a consequence of the oncogenic activation of particular LUAD driver genes.

We further tested whether *LRRK2* expression level was associated with an empirically derived, gene expression-based ‘tumor differentiation’ score, calculated for TCGA LUAD cohort by Chen et al. (Supplementary Table S1)^18^. The authors developed a gene signature from an alternate cohort of LUAD patients^22^, which had been histopathologically categorized by growth pattern into either less or more differentiated tumors. Less differentiated tumors (called ‘bronchial-derived’) exhibited invasive features with architectural destruction, while more differentiated tumors (called ‘bronchioloalveolar’) exhibited preservation of the lung architecture. Morphological indications of LUAD invasiveness are used to inform clinical prognosis^28^. Stratification of this tumor differentiation score by *LRRK2* and expression subtype status indicated that non-TRU type tumors were less differentiated (Dunn’s test, BH adjP < 0.0001)^17,19^, with *LRRK2*-low non-TRU tumors predicted to be the least differentiated (Spearman’s correlation coefficient − 0.59 with Holm’s adjP < 0.0001; Fig. 1F). Further, tumoral expression of established markers for key distal lung epithelial cell types (e.g., alveolar type (AT)1, AT2 and club cells)^29,30^ were significantly reduced in *LRRK2*-low non-TRU type tumors (Fig. 1G; Supplementary Fig. S3). To evaluate the cell type specific expression of *LRRK2* in lung, we took advantage of a recent publicly available single cell RNA-seq dataset (GSE131907^31^) that included 42,996 sequenced cells from the normal lung of 11 patients. Using cell type annotation reported by the authors, we determined that the vast majority of *LRRK2* expression in human lung occurs in AT2 cells and not in other known lung epithelial cells (Supplementary Fig. S4). Taken together, these results suggest that *LRRK2* reduction in LUAD likely occurs in AT2 cells, is associated with ongoing smoking, and may be associated with disease-related changes to peripheral lung architecture.

### Surfactant metabolism genes are tightly co-expressed with *LRRK2* in LUAD

**Figure 2:**
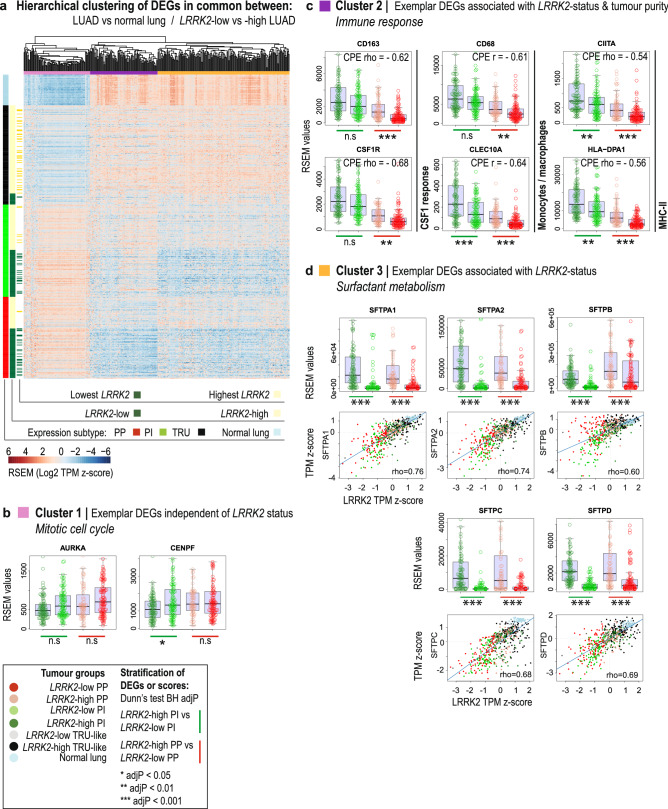
The transcriptional landscape of *LRRK2* repression in lung adenocarcinoma patients. (**A**) Heatmap depicting the hierarchical agglomerative clustering of genes differentially expressed in common between two biological contexts in TCGA LUAD tumors, each with a marked reduction of *LRRK2* expression: (1) across all tumors versus normal lung and (2) in the lowest *LRRK2* expressing tumors versus the highest *LRRK2* expressing tumors. Columns: n = 385 DEGs with absolute fold change of median RSEM value > 2; Rows: n = 59 normal lung and n = 517 LUAD tumors, ordered by tissue, *LRRK2* expression status and expression subtype. (**B**) Exemplar DEGs identified in Cluster 1, enriched for genes that act in mitotic cell cycle (Metascape algorithm; q < 0.05), stratified by tumor group (multicolour boxplots of exemplar gene expression; Dunn’s test BH adjP > 0.05). Tumor groups represent the combined sample status for *LRRK2* expression and non-TRU subtype. (**C**) Exemplar DEGs identified in Cluster 2, associated with tumor purity (consensus purity estimate or CPE; Spearman’s correlation coefficient ≥ 0.5 with Holm’s corrected P < 0.0001) and enriched for immune response genes (Metascape algorithm; q < 0.05), stratified by tumor group (multicolour boxplots of exemplar gene expression; Dunn’s test BH adjP < 0.01). (**D**) Exemplar DEGs identified in Cluster 3, enriched for genes that act in surfactant metabolism (Metascape algorithm; q < 0.05), stratified by tumor group (multicolour boxplots of exemplar gene expression; Dunn’s test BH adjP < 0.001) or plotted against *LRRK2* expression (multicolour scatterplots; Spearman’s correlation coefficient ≥ 0.6 with Holm’s corrected P < 0.0001; standardized RSEM Transcripts Per Million or TPM).

To profile the transcriptional landscape associated with a change in *LRRK2* expression, we compared the results of differential expression analyses for two biological contexts in LUAD with a marked decrease of *LRRK2* expression: in tumor (n = 517) versus normal lung (n = 59), and in tumors expressing the lowest (n = 50) versus highest deciles (n = 49) of *LRRK2* expression. A comparison of differentially expressed genes (DEGs) identified in each context revealed a considerable overlap of misexpressed genes (Supplementary Table S4), suggesting that altered cell processes associated with the transformation of normal lung dominated the transcriptional changes found in *LRRK2*-low tumors. Figure 2 depicts the hierarchical agglomerative clustering of the expression of a selection of DEGs, common to both contexts, plotted for the complete TCGA LUAD cohort and ordered by tissue, *LRRK2* expression status and expression subtype (n = 385 DE genes with absolute median RSEM value fold change > 2; Fig. 2A). To tease out DEGs associated with reduced tumoral *LRRK2* in an expression subtype-independent manner, we also stratified the expression of DEGs by *LRRK2* expression status in non-TRU subtype tumors only (Supplementary Table S4).

*LRRK2* status failed to be associated with the differential expression of most Cluster 1 cell cycle-related genes in non-TRU subtype tumors only (e.g., GO:0007346^32^; Fig. 2B), but was associated with a marked decrease of a number of Cluster 2 immune-related genes (e.g., GO:0019221^32^; Fig. 2C) and many Cluster 3 genes, enriched for genes that function in surfactant metabolism (e.g., Reactome R-HSA-5683826^33^; Fig. 2D). Moreover, Cluster 3 genes, including all surfactant genes (*SFTPA1*, *SFTPA2*, *SFTPB*, *SFTPC*, *SFTPD*) and various lamellar body-related genes (*ABCA3* and *LAMP3*), were strongly co-expressed with *LRRK2* (absolute Spearman’s correlation coefficient > 0.6 with Holm’s adjP < 0.0001), suggesting the tight transcriptional co-regulation of *LRRK2* with pathways known to be important for normal AT2 cell differentiation^34^ and function^35^. Therefore, differential expression results show that increased proliferation was associated with non-TRU type LUAD, regardless of *LRRK2* expression status, while *LRRK2* reduction was strongly associated with decreased surfactant gene expression, regardless of expression subtype. Indeed, applying principal component (PC) analysis, as an unbiased technique to reduce the dimensionality of the tumor versus normal transcriptional landscape of TCGA LUAD cohort, revealed that the top gene contributor to PC1—the component that explained the most variance—was *SFTPC*, while *LRRK2* was quantified as one of the top 3% of gene contributors to PC1 (Supplementary Fig. S5; gene loadings and sample scores in Supplementary Table S6).

While TCGA LUAD patient data were not available to assess changes to *LRRK2* and surfactant gene expression at the protein level, surfactant protein deficiency is known to be associated with smoking and lung disease in patients^36,37^, including cancer^38,39^. Given that LRRK2 knockout (KO) in rodents, or inhibition in primates, perturbs the morphology of lamellar bodies that package, store and secrete surfactant^6,40^, a pathological cycle may be triggered whereby ongoing smoking contributes to the repression of *LRRK2* and the surfactant pathway, while decreased LRRK2 further disrupts surfactant biogenesis and/or function. Pulmonary surfactant acts as a protective mechanical barrier, with surfactant proteins A and D directly modulating alveolar innate immunity^41^. Therefore, reduced *LRRK2* may lead to altered alveolar immunity that could undermine tumor immune surveillance^42^. Of note, *LRRK2*-low PP tumors from the TCGA LUAD cohort were predicted to have significantly lower mRNA levels of colony-stimulating factor-1 (CSF1) macrophage regulation genes, monocyte and macrophage marker genes, as well as major histocompatibility complex II (MHC-II) pathway genes (Fig. 2C). Further, Cluster 2 immune-related genes—but not *LRRK2* (Spearman’s correlation coefficient = − 0.14 with Holm’s adjP < 0.0001)—were strongly negatively associated with tumor purity (consensus purity estimate^43^; Spearman’s correlation coefficient ≤ − 0.5 with Holm’s corrected P < 0.0001) (Supplementary Table S4), predicting that their expression depended to a large extent on non-tumor cells. To corroborate this interpretation, we mined the above mentioned recent public dataset of single cell RNA-seq from normal human lung (GSE131907^31^). Using the cell type annotation reported by the authors, we confirmed that the myeloid-related genes displayed in Fig. 2C were expressed in this new dataset mainly in myeloid cells, while the MHC-II gene *HLA-DPA1* was expressed in some epithelial cells, as well as in various white blood cells (Supplementary Fig. S6). Therefore, we predict that the changes in myeloid-related gene expression we observed in *LRRK2*-low versus -high PP tumors (Fig. 2C) represented changes in tumor infiltration by myeloid cells, whereas the changes in expression of MHC-II genes, such as *HLA-DPA1*, may have occurred in both tumor cells and various immune cells.

While the mechanisms that link surfactant dysfunction to lung tumorigenesis remain unknown, robust associations exist between smoking history, surfactant defects, immunosuppression and lung cancer. Low *LRRK2* non-TRU tumors may constitute a LUAD tumor subset that reflects increased surfactant dysfunction and altered tumor immunity.

### *LRRK2*-low LUAD shares patterns of differential gene expression with postnatal developing lung, during alveolarization

A literature search of genes misexpressed in *LRRK2*-low tumors identified a recent study of lung development in mice^44^, in which, just as in TCGA LUAD cohort, cell cycle genes and surfactant metabolism genes were reported to be inversely or co-expressed with *LRRK2* (respectively), during postnatal alveolarization. Alveolarization refers to the postnatal differentiation of alveoli from prenatal saccules. To assess whether DEGs in *LRRK2*-low tumors overlapped significantly with genes found to be differentially expressed in developing mouse lung, we converted the human DEGs to their mouse gene orthologs^45,46^ (Supplementary Table S5), and employed a Monte Carlo permutation simulation (n = 100,000 repetitions; Bonferroni corrected empirical P-value) to generate an empirical distribution of the overlap that would occur by chance^47^. Table 1 summarizes the significance of overlap between DEGs in *LRRK2*-low LUAD tumors and genes differentially expressed between adjacent developmental stages in mice. The observed intersection of DEGs in *LRRK2*-low tumors and a particular developmental time point—between two stages of gene expression-defined secondary alveolar septation (i.e. postnatal days P0-P3 (called ALV1) versus P4-P7 (called ALV2)^44^)—was substantially greater than expected by chance: 4.5 times the expected number of differentially increased genes (n = 208 genes) and 2.8 times the expected number of differentially decreased genes (n = 252 genes) overlapped between studies. Gene set enrichment analysis revealed that shared increased DEGs function in cell cycle pathways (e.g., R-HSA-1640170), while shared decreased DEGs function in surfactant metabolism (e.g., R-HSA-5683826) (^33,48^; Supplementary Table S5).

**Table 1:** Overlap of DEGs identified in the lowest *LRRK2*-expressing TCGA LUAD tumors with DEGs identified in mouse lung development.

Developmental time points	Mouse lung development DEG list	Gene count ^a^	LUAD DEG list	Gene count	Observed gene intersection^b^	Simulated gene intersection (min–max)	Empirical P-value^c^	Observed > Simulated intersection?^d^
E11.5–12.5 vs E13.5-E15.5	EMB vs PSG ↑	1335	Lowest *LRRK2* vs Highest *LRRK2* (↑)	1302	88	32–102	0.0014	No
E11.5–12.5 vs E13.5-E15.5	EMB vs PSG ↓	190	Lowest *LRRK2* vs Highest *LRRK2* (↓)	2199	27	2–35	0.0018	No
E13.5-E15.5 vs E16.5-E17.5	PSG vs CAN ↑	1021	↑	1302	40	23–87	0.91	No
E13.5-E15.5 vs E16.5-E17.5	PSG vs CAN ↓	757	↓	2199	45	32–96	0.99	No
E16.5-E17.5 vs E18.5_E19.5	CAN vs SAC ↑	853	↑	1302	31	18–70	0.95	No
E16.5-E17.5 vs E18.5_E19.5	CAN vs SAC ↓	806	↓	2199	26	36–102	1.00	No
E18.5_E19.5 vs P0-P3	SAC vs ALV1 ↑	855	↑	1302	45	17–70	0.25	No
E18.5_E19.5 vs P0-P3	SAC vs ALV1 ↓	594	↓	2199	59	23–77	0.055	No
**P0-P3 vs P4-P7**	**ALV1 vs ALV2 ↑**	**473**	**↑**	**1302**	**208**	**6–46**	** < 0.0001**	**Yes**
**P0-P3 vs P4-P7**	**ALV1 vs ALV2 ↓**	**692**	**↓**	**2199**	**252**	**27–89**	** < 0.0001**	**Yes**
P4-P7 vs P9-P12	ALV2 vs ALV3 ↑	273	↑	1302	15	0–30	0.25	No
P4-P7 vs P9-P12	ALV2 vs ALV3 ↓	371	↓	2199	25	11–57	0.82	No
P9-P12 vs P13-P18	ALV3 vs ALV4 ↑	592	↑	1302	18	10–56	0.98	No
**P9-P12 vs P13-P18**	**ALV3 vs ALV4 ↓**	**623**	**↓**	**2199**	**85**	**24–84**	** < 0.0001**	**Yes**
P13-P18 vs P21-P56	ALV4 vs MAT ↑	836	↑	1302	63	16–70	0.00030	No
**P13-P18 vs P21-P56**	**ALV4 vs MAT ↓**	**643**	**↓**	**2199**	**139**	**27–83**	** < 0.0001**	**Yes**

*↑/↓* increased/decreased, *EMB* embryonic, *PSG* pseudoglandular, *CAN* canalicular, *SAC* saccular, *ALV1-4* alveolarization stages 1–4, *MAT* mature lung.

^a^Gene count is defined as the count of differentially increased or decreased genes, identified in lung development stage comparisons by Beauchemin et al.^44^ or in this study.

^b^Observed gene intersection was calculated as the count of shared DEGs, per comparison.

^c^Empirical p-value was calculated per gene intersection as p = (b + 1)/(m + 1), where: b was the number of times a simulated gene intersection was ≥ the observed gene intersection and m was the number of permutations (m = 100,000) (Bonferroni adjP < 0.003 for 16 comparisons).

^d^’yes’ and bold rows indicate that the observed gene intersection was greater than the maximum simulated gene intersection.

Morphological characterizations of postnatal lung development in rodents have documented an exponential increase in alveoli number that occurs between postnatal days P3 and P14^49–51^, where new growth of septal membrane outpaces overall lung growth on days P4 to P7^52^. Alveoli have also been shown to increase dramatically in human lung during the first 2 years of life, followed by a slow but continuous increase into adolescence^53^. It is possible that postnatal alveolar development mechanisms remain available in adult lung and could be co-opted by lung cancer cells—perhaps during alveolar regeneration following repetitive lung injury^54^, or by smoking-related oxidative stress that mimics the transition from a hypoxic in utero environment to the 21% oxygen of ambient air^55^. Based on shared patterns of differential gene expression, we propose that aberrant activation of a developmental program of alveolar growth and differentiation contributes to the hyperproliferation and surfactant defects predicted in our study, for *LRRK2*-low non-TRU type LUAD.

### LRRK2 knockout increases tumor initiation in a murine model of early lung cancer, induced by a carcinogen present in cigarette smoke

**Figure 3:**
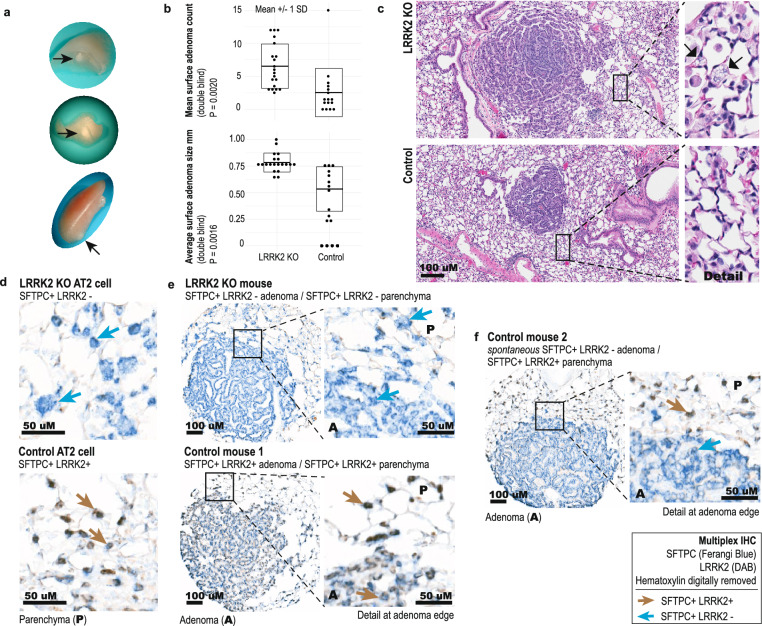
LRRK2 knockout increases SFTPC-positive tumor initiation in a murine model of urethane-induced, early stage lung cancer. (**A**) Representative visible adenomas that developed in the lungs of both LRRK2 knockout (KO) mice and wild type (WT) strain mates, following urethane treatment. (**B**) Double blind analysis of adenoma counts and size (n = 16 WT and n = 20 LRRK2 KO animals, with n = 40 WT and n = 130 LRRK2 KO surface lesions). (**C**) Representative H&E stained sections of adenomas (20× magnification; transverse section; n = 16 WT and n = 20 LRRK2 KO animals, with n = 40 WT and n = 130 LRRK2 KO surface lesions). (Inset) Detailed view of representative vacuolated cells (arrows) found in LRRK2 KO lung only. (**D**) Representative images of multiplexed IHC staining for colocalization of alveolar type II (AT2)-specific cell marker SFTPC (Ferangi blue chromogen) and LRRK2 (3,3′-Diaminobenzidine (DAB) chromogen), in LRRK2 KO (top) and WT (bottom) mouse lung parenchyma (n = 7 WT mice with 14 lesions; n = 15 KO mice with 25 lesions). (**E**) Representative images of multiplexed IHC staining for SFTPC and LRRK2, in adenomas that developed in the lungs of both LRRK2 KO (top) and WT (bottom) control mice, following urethane treatment (n = 7 WT mice with 14 lesions; n = 15 KO mice with 25 lesions). (Inset) detailed view of staining at adenoma edge and adjacent lung parenchyma. (**F**) Representative images of multiplexed IHC staining for SFTPC and LRRK2, for adenomas that developed in the lungs of WT control mice following urethane treatment, showing spontaneous reduction of LRRK2 positivity. Representative of 4 of 14 lesions from n = 7 WT control mice. (Inset) AT2 cells in the adjacent lung parenchyma remained double positive for SFTPC and LRRK2.

Given the association of reduced *LRRK2* with current smoking and decreased patient survival, increased genome instability and less differentiated non-TRU tumor types, we hypothesized that *LRRK2* has tumor suppressive effects in carcinogen-exposed lung tissue. To assess whether LRRK2 KO increases adenoma multiplicity and/or size, 6- to 8-week-old C57BL/6-Lrrk2^tm1Mjfa^ null mice of both sexes, homozygous for a deletion in Exon 41 of the kinase domain, and wild type (WT) strain mates received 10 weekly intraperitoneal injections of urethane (1 g/kg diluted in PBS), and were sacrificed at 26 weeks post‐first injection^56^ (Fig. 3). WT mice produced an average of 2.53 ± 0.91 (mean ± SEM) visible adenomas (n = 16 mice; 95% CI 0.702–4.36 including outlier animal), while LRRK2 KO mice produced an average of 6.53 ± 0.75 adenomas per animal (n = 20 mice; 95% CI 5.02–8.03) (Welch’s two-sample t-test, t(30.94) = − 3.38, 95% CI − 6.41–1.58, P = 0.0020, d = 1.14) (Fig. 3A–C). LRRK2 KO adenomas were larger on average, per animal: 0.53 ± 0.06 mm in WT (95% CI 0.41–0.65) and 0.78 ± 0.02 mm in LRRK2 KO (95% CI 0.74–0.82) (n = 40 WT and 130 KO surface lesions; Welch's two-sample t-test, t(13.46) = − 3.93, 95% CI − 0.39–0.11, P = 0.0016, d = 1.72) (Fig. 3A–C). No sex-specific differences were found. IHC staining showed an equivalent percent of Ki-67 positive adenoma cells between genotypes, suggesting that LRRK2 KO may have facilitated tumor development at an earlier time point; granted, given the long latency of lesion development in this model, we cannot rule out that differences in proliferation rate existed closer to treatment.

To confirm LRRK2 expression in AT2 cells in our model, we applied multiplexed IHC staining for AT2 cell-specific marker SFTPC (Ferangi blue chromogen) with LRRK2 (3,3′-Diaminobenzidine chromogen) to determine colocalization (Fig. 3D). LRRK2 colocalized almost exclusively with SFTPC in WT mouse lung, in both adenomas and parenchyma (Fig. 3E), locating LRRK2′s main functional relevance in this model to AT2 cells, a predominant cell type of origin in LUAD^7^. Further, SFTPC + LRRK2-AT2 cells appeared less punctate with SFTPC IHC (Fig. 3D top), compared to SFTPC + LRRK2 + AT2 cells (Fig. 3D bottom), confirming that LRRK2 loss perturbs AT2 cell morphology and could contribute to AT2 cell dysfunction. Of note, 4 of 14 adenomas that developed in the lungs of WT control mice (n = 7) showed a reduction of LRRK2 positivity, while adjacent lung parenchyma remained double positive for SFTPC and LRRK2 (Fig. 3F). Therefore, LRRK2 KO not only increased tumor formation, but LRRK2 expression appeared reduced in some WT AT2 cells, in response to tumor-related changes triggered by urethane.

## Conclusions

In summary, marked *LRRK2* reduction was associated with tumors predicted to have altered distal lung architecture, dysfunctional surfactant metabolism, hyperproliferation and potentially altered innate tumor immunity—in patients who continued to smoke and ultimately suffered worse survival. While a retrospective study of associations identified in patients cannot determine causation, we were able to show that systemic LRRK2 KO increased tumor initiation and size in carcinogen-driven early lung cancer in mice, providing orthologous evidence that LRRK2 reduction is not merely a passenger aberration but a link between environmental insult and potential AT2 cell transformation.

This is the first study to demonstrate a tumor suppressive role for LRRK2 in carcinogen-induced distal lung cancer, and to potentially identify a Parkinson’s patient population (i.e. current smokers) for whom long-term LRRK2 inhibition may require additional monitoring. Further studies that identify the mechanisms by which LRRK2 reduction promotes LUAD tumorigenesis have the potential to benefit cancer patients, and to inform the design of LRRK2 inhibition strategies in Parkinson’s disease that mitigate potential pulmonary-related adverse effects.

## Methods

### TCGA data and associated molecular tumor features

The following data were downloaded from the Broad Institute TCGA Genome Data Analysis Center: Level 3 TCGA LUAD clinical data (10.7908/C19P30S6), Level 3 TCGA LUAD mutation calls (10.7908/C11G0KM9), Level 4 somatic DNA alterations (SNP6 GISTIC2 copy number analysis (10.7908/C1348JSB), Level 4 MutSig 2CV version 3.1 mutation analysis (10.7908/C17P8XT3)), Level 3 TCGA LUAD RNA-seq data (10.7908/C11G0KM9). Survival data for the LUAD cohort were obtained from the TCGA Pan-Cancer Clinical Data Resource^16^. Additional molecular tumor features predicted from RNA-seq data included scores for immune cell infiltration and regulation^57^ and tumor differentiation status^18^. To test for associations, *LRRK2* gene abundance was dichotomized using the X-tile cohort separation algorithm^15^. Gene expression subtypes were assigned to TCGA LUAD tumor samples as previously published^17^.

### Cutpoint derivation to define *LRRK2* expression status of TCGA LUAD patients

To test for associations between *LRRK2* expression status and various clinical phenotypes and molecular tumor features, *LRRK2* gene abundance (quantile normalized RSEM read counts) was dichotomized using the X-tile cohort separation algorithm (RRID:SCR_005602)^15^. X-tile determines a cutpoint by optimizing cohort correlation with survival data, which was achieved in this study by minimizing the P-value defined by Kaplan–Meier analysis of 5-year overall survival outcomes (n = 460 patients, with tumor samples having greater than 60% tumor nuclei) and the Log-rank test (Monte Carlo cross-validation with n = 10,000 repetitions P < 0.05). A cutpoint of 588.92 quantile normalized RSEM was determined for *LRRK2* and used in downstream analyses.

### Somatic mutation and copy number alteration stratified by *LRRK2* cutpoint and expression subtype

Scores for genome-wide mutation and copy number load were calculated as previously published^58^. The mutational load was calculated based on exome sequencing data, by normalizing the count of all somatic mutations per tumor to an approximate protein-coding exome size of 30 megabases. The copy number load was calculated per tumor as the number of nucleotides affected by copy number alteration (at an absolute segmented value greater than 0.3), normalized to the total number of nucleotides contained in all segments identified for that tumor.

Non-synonymous somatic mutations—missense, splice site, frameshift or other non-synonymous—reported for significantly mutated genes (SMGs; n = 52 genes) identified by the TCGA MutSig 2CV v3.1 pipeline (q < 0.025) (RRID:SCR_010779)^21^ were tested for association with *LRRK2* cutpoint and expression subtype status (pairwise Fisher’s exact test BH adjP < 0.05). Tumor stratification served as row variable and mutation status was binarized as altered (value = 1) or unaltered (value = 0). Gene-level TCGA LUAD copy number data was filtered for either unaltered or high-level copy number alterations. High-level thresholds were calculated by TCGA on a per sample basis as the maximum (for amplifications) or minimum (for deletions) median arm-level copy number alteration^21^. Genes altered in at least 5% of tumors with high-level copy number alterations were tested for association with tumors grouped by combined status for *LRRK2* cutpoint and expression subtype (n = 2458 genes; pairwise Fisher’s exact test with BH adjP < 0.05). After identifying gene-level SCNA associations per tumor group, genes found to be differentially copy number altered in *LRRK2*-high versus -low tumors within a non-TRU subtype were interpreted as being associated with a change in *LRRK2* status (i.e. CNAs differentially altered in *LRRK2*-high PP vs *LRRK2*-low PP or *LRRK2*-high PI vs *LRRK2*-low PI). Genes found to be differentially copy number altered between various subtypes within *LRRK2*-high tumors were interpreted as being associated with a change in expression subtype, independent of *LRRK2* expression status (i.e. *LRRK2*-high TRU vs *LRRK2*-high PI, *LRRK2*-high TRU vs *LRRK2*-high PP, *LRRK2*-high PI vs *LRRK2*-high PP). Genes found to be differentially copy number altered in *LRRK2*-high versus -low tumors within a non-TRU subtype but not between expression subtypes within *LRRK2*-high tumors were interpreted as the most likely to be associated with a change in *LRRK2* level, independent of subtype.

### Assignment of molecular expression subtypes

To assign LUAD gene expression subtypes^17,19,21^ to TCGA LUAD tumor samples (n = 515 tumors) as previously published, quantile normalized RSEM gene expression data were Log2 transformed, median centered by gene and compared by Pearson correlation to the published nearest centroid classifier^17^. Input genes were limited to the 489 of 506 classifier genes present in the TCGA data set and subtype was assigned using the maximum correlation coefficient. Subtype calls were benchmarked against published subtype assignments by TCGA Research Network^21^ on a subset of the TCGA LUAD cohort (n = 230 tumors), which found 93.9% of calls to be identical—a result in line with a recent assessment of the classification stability for this classifier, which found that typically < 10% of samples switched gene expression subtype when perturbations to the input data (e.g., missing genes, missing samples, differences in gene centering or similarity metric) were introduced ^59^.

### Differential expression analyses and gene set enrichment

Quantile normalized RSEM (RID:SCR_013027) read counts were further normalized for library size using the trimmed mean of M-values (TMM) method implemented in edgeR version 3.22.3 (calcNormFactors function; RRID:SCR_012802); normalized log-counts per million were weighted using the variance modelling at the observation-level method prior to the Limma analysis pipeline version 3.34.5 (RRID:SCR_010943). Differentially expressed (DE) genes identified by Limma were filtered for a false discovery rate < 0.05 (BH adjusted), an average log expression greater than 1, and an absolute log fold change greater than 0.6. DE analysis was performed between the following sample stratifications: tumor versus adjacent normal lung, tumors expressing the lowest decile of *LRRK2* gene abundance (*LRRK2*-D1) versus those expressing the highest decile of *LRRK2* gene abundance (*LRRK2*-D10), *LRRK2*-high versus -low PI tumors, *LRRK2*-high versus -low PP tumors, as well as *LRRK2*-high versus -low non-TRU type tumors (PI and PP subtypes combined). Genes DE between *LRRK2*-D1 and *LRRK2*-D10 tumors were compared to genes DE in tumors versus normal lung—e.g. a list of genes differentially increased in *LRRK2*-D1 versus -D10 tumors was compared to the list of genes differentially increased in all tumors versus normal lung—producing the following input gene lists for gene set expression analysis (GSEA) by the Metascape algorithm^48^ (q < 0.05; RRID:SCR_016620; http://metascape.org): (1) common increased DE (in all tumors and *LRRK2*-D1 tumors), (2) common decreased DE (in all tumors and *LRRK2*-D1 tumors), (3) and (4) opposing increased or decreased DE in *LRRK2*-D1 tumors (compared to all tumors), (5) and (6) uniquely increased or decreased DE in *LRRK2*-D1 tumors (compared to all tumors), or (7) and (8) uniquely increased or decreased DE in all tumors (compared to *LRRK2*-D1 tumors). Genes DE between *LRRK2*-D1 and -D10 tumors found to be further DE with a change in *LRRK2* expression level within tumors of the same expression subtype—i.e. *LRRK2*-high versus -low PP tumors, *LRRK2*-high versus -low PI tumors or *LRRK2*-high versus low non-TRU type tumors—were interpreted as having DE associated with *LRRK2* expression status. To identify genes potentially co-expressed with *LRRK2*, a Spearman’s correlation coefficient was calculated for the expression of each gene in the genome and *LRRK2* (n = 515 LUAD tumors; absolute Spearman’s correlation coefficient > 0.3, BH adjP < 0.05). Exploratory hierarchical agglomerative clustering was performed for a filtered set of genes, DE in both tumors versus normal lung and *LRRK2*-D1 versus -D10 tumors (absolute median RSEM fold change between respective sample groups > 2), using standardized Log2 transformed RSEM Transcripts Per Million and complete linkage with Euclidean distance (hclust function in ‘stats’ package version 3.4.3).

### Predicted tumor purity and tumor differentiation status

The consensus measurement of tumor purity estimate (CPE) was obtained from supplementary material published by Aran et al., who estimated purity for more than 10,000 samples across 21 cancer types from TCGA by amalgamating the results of four methods that relied on separate data types: ESTIMATE (expression profiles of immune and stromal genes), ABSOLUTE (somatic copy-number data), LUMP (methylation of immune-specific CpG sites) and image analysis of haematoxylin and eosin slides^43^. To identify gene expression impacted by tumor purity, a Spearman’s correlation coefficient was calculated for the expression of each gene in the genome and the CPE, for all TCGA LUAD tumor samples (absolute Spearman’s correlation coefficient > 0.3 and Holm’s adjP < 0.05).

A gene expression-based tumor differentiation score was obtained from the supplementary material of Chen et al.^18^. The differentiation score was calculated as a standardized T-score that compared the mean expression of genes associated with more tumor differentiation to the mean expression of genes associated with less tumor differentiation, per TCGA LUAD tumor sample. The larger the absolute value of the score, the larger the difference in expression between gene sets; therefore, a positive score reflected increased expression of genes associated with less differentiated tumors relative to genes associated with more differentiated tumors, while a negative score indicated the reverse.

### Human and mouse gene homology

Gene homology between human and mouse was used to compare transcriptional profile results from this study to a previous study of DE mouse lung development genes identified in fetal and neonatal mouse lungs at 26 time points, termed the murine Developing Lung Characteristic Subtranscriptome (mDLCS)^44^. *LRRK2* was identified as a member of the mDLCS. Mouse Gene Identifiers (MGI) for mDLCS genes and their direction of DE between 9 sequential developmental stages—four prenatal stages (embryonic, pseudoglandular, canalicular and saccular), four post natal stages of alveolarization (ALV1, ALV2, ALV3, ALV4) and a final homeostatic stage (maturity)—were obtained from the supplementary material of Beauchemin and colleagues^44^. A comprehensive gene homology resource for human-mouse orthologs was downloaded using the HGNC Comparison of Orthology Predictions (HCOP) tool (https://www.genenames.org/tools/hcop/)^45,46^, which amalgamates orthology predictions between human and mouse from 12 curated databases (eggnog, Ensembl, HGNC, HomoloGene, Inparanoid, OMA, OrthoDB, NCBI Gene Orthology, OrthoMCL, Panther, PhylomeDB, TreeFam). The resource was filtered for human-mouse orthology predictions that had support from at least two databases and where each human-mouse orthology pair was annotated with unique HUGO Gene Nomenclature Committee (HGNC) and Mouse Genome Informatics (MGI) identifiers. If one gene mapped to many orthologs, each ortholog was included. The final human-mouse orthology reference used in this study included: 18,650 human genes and 19,613 mouse genes, with 26,855 unique HGNC-MGI identifier pairs—2398 human genes mapped to multiple mouse orthologs (a total of 4285 mouse genes), while 2668 mouse genes mapped to multiple human orthologs (a total of 3231 human genes). DE genes identified in lung cancer in this study and DE mouse development genes identified in the Beauchemin study were included for comparison if they had an ortholog predicted by HCOP in our curated human-mouse orthology reference: 96.4% of human genes identified as DE in this study had at least one mouse ortholog (3501 of 3633 genes), while 92.4% of mouse genes identified as DE by Beauchemin et al. had at least one human ortholog (5107 of 5,527 genes).

### Significance of gene list overlap by Monte Carlo permutation test

After converting human genes identified as DE in this study to their predicted mouse orthologs using the above described human-mouse orthology reference, the overlap between genes found to be differentially increased in *LRRK2*-low versus -high LUAD tumors were compared with genes differentially increased between neighbouring developmental stages during lung development in the Beauchemin study (i.e. embryonic versus pseudoglandular, pseudoglandular versus canalicular, etc.); a similar strategy was used for genes that were differentially decreased in *LRRK2*-low versus -high LUAD tumors and during mouse development, for a total of 16 comparisons (Table 1). To test the significance of gene list overlap, a Monte Carlo permutation simulation without replacement^47^ (n = 100,000 repetitions) was applied to generate an empirical distribution of the overlap that would occur by chance between two lists of genes with variable lengths, given a common pool of 26,855 possibilities—the number of unique HGNC-MGI identifier pairs found in the human-mouse orthology reference, from which the DE human lung cancer genes and DE mouse development genes were constrained to be drawn. An empirical P-value was calculated for each overlap as p = (b + 1)/(m + 1), where b was the number of times a simulated gene list overlap was greater than or equal to the observed number of genes found to overlap and m was the number of permutations^47^. P-values were further corrected for multiple comparisons (Bonferroni corrected P < 0.003).

### In vivo study

The care, housing and use of animals was performed in accordance with the Canadian Council on Animal Care Guidelines. All animal experiments were approved by the Animal Care Committee of the University of British Columbia (A14-0290). Further, all animal experiments were performed in accordance with the relevant guidelines and regulations approved by this committee, as well as with the ARRIVE guidelines (https://arriveguidelines.org/). C57BL/6-Lrrk2^tm1Mjfa^ mice, which have Exon 41 of the kinase domain deleted, were a generous gift by Dr. Matthew J Farrer^60^ and were previously deposited at Jackson laboratories (RRID:IMSR_JAX:012,444). LRRK2 KO and WT comparison lines were both derived from heterozygote breeding pairs from the C57BL/6-Lrrk2^tm1Mjfa^ strain. To obtain sufficient animals for the treatment study, multiple different littermate homozygous WT or homozygous LRRK2 KO breeding pairs and trios were maintained for 1 to 3 generations and used to generate 20 age-matched animals per genotype (10 males and 10 females). LRRK2 KO and WT strain mates received 10 weekly intraperitoneal (i.p.) injections of urethane (1 g/kg diluted in PBS) beginning at 6–8 weeks old^56^. The health status of the animals was monitored following an established standard operating procedure. In particular, signs of ill health were based on body weight loss, change in appetite, and behavioral changes such as altered gait, lethargy, and gross manifestations of stress. When signs of severe ill health were present, which occurred in 4 of 20 WT animals, animals were terminated (sodium pentobarbital overdose followed by CO2 asphyxiation) for humane reasons. Surviving mice were euthanized 26 weeks post-first treatment by an overdose (240 mg/kg i.p.) of freshly prepared sodium pentobarbital, followed by exsanguination and tracheal instillation of 10% neutral buffered formalin into lungs, which were then collected and fixed by overnight immersion. Full necropsies were completed on all mice to assess whether there were gross changes in tissue/organ appearance. Lung lobes were separated, and each lobe examined under dissection microscope by two researchers blind to the study design. Surface lesions greater than 0.2 mm were counted and measured using digital micro calipers. Lungs were then paraffin embedded, with each of the five lung lobes per animal arranged in a standard configuration. A single transverse, 5 μm thick tissue section per animal was stained by hematoxylin and eosin and examined by the study pathologist and a second experienced researcher, both blind to study design, and micro lesions (classified as adenomas as outlined by the Mouse Models of Human Cancers Consortium^61^) were counted per animal. All counts and measurements were averaged between readers and differences between groups were tested by Welch’s two-sample T test.

### Multiplex immunohistochemistry

To construct a tissue microarray, 0.6 mm tissue cores containing adenomas and adjacent lung parenchyma from each animal in the study were prepared with a tissue arrayer instrument (Beecher Instruments, Sun Prairie, WI) across two blocks. The following protocol for multiplex IHC was adapted from previous work^62^. All reagents used for immunohistochemistry were from Biocare Medical (Pacheco, CA) unless otherwise stated. Slides of formalin-fixed, paraffin embedded tissue were incubated overnight at 37 °C, then deparaffinized and rehydrated through xylene and graded alcohols. Antigen retrieval was performed using Rodent Decloaker in a Biocare decloaking chamber at 110 °C for 30 min. Slides were then rinsed with water and loaded into the Biocare Intellipath FLX autostainer. Slides were blocked with Hydrogen Peroxide Blocking Reagent (ab64218, Abcam) and Rodent Block for 10 min and 30 min respectively, before adding a cocktail of SFTPC (1/3000, clone EPR19839, ab211326, Abcam) and LRRK2 (1/600, clone N241A/34, 75–253, Antibodies Incorporated^63^) in Da Vinci Green diluent for 30 min at room temperature. Following a wash step, Mouse on Mouse HRP Polymer (ab127055, Abcam) was added for 15 min at room temperature, then Mach2 Rabbit AP-Polymer for 30 min, prior to antigen detection with IP Ferangi Blue chromogen for 8 min and after, IP DAB chromogen for 5 min. Finally, CAT hematoxylin at a 1/5 dilution was added for five minutes and slides were washed, air-dried and coverslipped with Ecomount coverslipping medium.

Multispectral images (20X magnification) of entire cores were collected using the PerkinElmer Vectra system. Quantification was performed using inform Advanced Image Analysis Software (PerkinElmer). Four separate visual algorithms were used to train inform to segregate cores into adenoma and adjacent lung parenchyma tissue. Each algorithm was run on images from all cores and cells within each tissue segment were binned into single positive, double positive and negative stain categories. Algorithm performance was validated manually (per algorithm) by visually comparing algorithm cell classification results to the captured images of each core. To calculate frequency, cell counts per stain category, per tissue segment, were averaged across all algorithms and divided by the total counts of cell nuclei per tissue segment; frequency was presented as a percentage of all tissue segment cells. Median frequency and confidence intervals were calculated using Efron's nonparametric bias-corrected and accelerated (BCa) bootstrap method per group (n = 10,000 repetitions; groupwiseMedian function in ‘rcompanion’ package version 1.13.2).

### LRRK2 antibody validation by western blot analysis

Snap frozen mouse lung tissue was minced and transferred to Lysing Matrix M tubes (MP Biomedical Cat#6923-050) containing a single zirconium coated ceramic bead (MP Biomedical Cat# 6540-034) and 300uL of RIPA Lysis Buffer (Santa Cruz Biotech Cat#sc-24948A). Samples were then homogenized using an MP Biomedical FastPrep-24 Classic bead beating grinder and lysis system at 6 m/s for 30 s. The tissue homogenate was transferred to a fresh tube and centrifuged at 13,000 RPM for 20 min at 4 °C. The supernatant was transferred to a fresh tube and protein concentration was determined using a Pierce BCA Protein Assay Kit (Thermo Scientific Cat# PI23227). Sixty ug of protein from LRRK2 WT and KO mouse lung samples were resuspended in BOLT LDS sample buffer (Invitrogen Cat# B0007), with BOLT sample reducing agent (Invitrogen Cat# B0009). These were then loaded onto a NuPAGE 3–8% tris–acetate gel (Invitrogen Cat#EA0376BOX) for gel electrophoresis using MOPS Buffer (Invitrogen Cat#B0001), for 50 min at 150 V, prior to transfer onto a PVDF membrane (Bio-Rad Cat#162-0177). The blot was blocked in 5% milk in PBS Tween-20 for 1 h at room temperature, then probed with 1/500 LRRK2 Ab (Antibodies Inc Cat# 75-253) overnight at 4 °C. The blot was then probed with 1/200 Veriblot secondary Ab (Abcam Cat# ab131366) for 2 h at room temperature. Three washes of 15 min each were completed at room temperature. Clarity ECL Reagent (Bio-Rad Cat# 170-5061) was used for immunodetection (Supplementary Fig. S7).

### Statistical information

Unless otherwise stated, statistical calculations were performed using R (version 3.4.3; www.R-project.org).

## Supplementary Information

Supplementary Table S1.

Supplementary Table S2.

Supplementary Table S3.

Supplementary Table S4.

Supplementary Table S5.

Supplementary Table S6.

Supplementary Information.

## Data Availability

This study is based in part on data generated by the TCGA consortium (https://www.cancer.gov/tcga). All TCGA LUAD patient data used in the current study are publicly available through the Broad Institute’s TCGA GDAC Firehose (http://gdac.broadinstitute.org/), using the digital object identifiers cited in “Methods”. The authors declare that all other data supporting the findings of this study are available within the paper or its supplementary information files.

## Abbreviations

AT2: Alveolar type II cells
DEG: Differentially expressed gene
DSS: Disease-specific survival
KO: Knockout
LRRK2: Leucine rich repeat kinase 2
LUAD: Lung adenocarcinoma
non-TRU: Non-terminal respiratory unit
OS: Overall survival
PI: Proximal-inflammatory
PP: Proximal-proliferative
TCGA: The Cancer Genome Atlas
TRU: Terminal respiratory unit

## References

[1] World Health Organization. Cancer key facts. https://www.who.int/news-room/fact-sheets/detail/cancer (2018).

[2] Alderton GK (2014). Tumour evolution: Clonal ancestry in lung cancer. Nat. Rev. Cancer.

[3] Lebovitz CB (2015). Cross-cancer profiling of molecular alterations within the human autophagy interaction network. Autophagy.

[4] Feng DD, Cai W, Chen X (2015). The associations between Parkinson’s disease and cancer: The plot thickens. Transl. Neurodegener..

[5] Kluss JH, Mamais A, Cookson MR (2019). LRRK2 links genetic and sporadic Parkinson’s disease. Biochem. Soc. Trans..

[6] Fuji RN (2015). Effect of selective LRRK2 kinase inhibition on nonhuman primate lung. Sci. Transl. Med..

[7] Rowbotham SP, Kim CF (2014). Diverse cells at the origin of lung adenocarcinoma. Proc. Natl. Acad. Sci..

[8] Sardi SP, Cedarbaum JM, Brundin P (2018). Targeted therapies for Parkinson’s disease: From genetics to the clinic. Mov. Disord..

[9] Baptista MAS (2020). LRRK2 inhibitors induce reversible changes in nonhuman primate lungs without measurable pulmonary deficits. Sci. Transl. Med..

[10] Rugbjerg K, Friis S, Lassen CF, Ritz B, Olsen JH (2012). Malignant melanoma, breast cancer and other cancers in patients with Parkinson’s disease. Int. J. Cancer.

[11] Gallo V (2018). Exploring causality of the association between smoking and Parkinson’s disease. Int. J. Epidemiol..

[12] Agalliu L (2019). Cancer outcomes among Parkinson’s disease patients with leucine rich repeat kinase 2 mutations, idiopathic Parkinson’s disease patients, and nonaffected controls. Mov. Disord..

[13] Allegra R, Tunesi S, Cilia R, Pezzoli G, Goldwurm S (2014). LRRK2-G2019S mutation is not associated with an increased cancer risk: A kin-cohort study. Mov. Disord. Off. J. Mov. Disord. Soc..

[14] Ruiz-Martínez J (2014). Prevalence of cancer in Parkinson’s disease related to R1441G and G2019S mutations in LRRK2. Mov. Disord. Off. J. Mov. Disord. Soc..

[15] Camp RL, Dolled-Filhart M, Rimm DL (2004). X-tile: A new bio-informatics tool for biomarker assessment and outcome-based cut-point optimization. Clin. Cancer Res..

[16] Liu J (2018). An integrated TCGA pan-cancer clinical data resource to drive high-quality survival outcome analytics. Cell.

[17] Wilkerson MD (2012). Differential pathogenesis of lung adenocarcinoma subtypes involving sequence mutations, copy number, chromosomal instability, and methylation. PLoS ONE.

[18] Chen F (2017). Multiplatform-based molecular subtypes of non-small-cell lung cancer. Oncogene.

[19] Hayes DN (2006). Gene expression profiling reveals reproducible human lung adenocarcinoma subtypes in multiple independent patient cohorts. J. Clin. Oncol..

[20] Bryant CM (2010). Clinically relevant characterization of lung adenocarcinoma subtypes based on cellular pathways: An international validation study. PLoS ONE.

[21] Collisson EA (2014). Comprehensive molecular profiling of lung adenocarcinoma. Nature.

[22] Beer DG (2002). Gene-expression profiles predict survival of patients with lung adenocarcinoma. Nat. Med..

[23] The Cancer Genome Atlas Research Network (2014). Comprehensive molecular profiling of lung adenocarcinoma. Nature.

[24] Faruki H (2017). Lung adenocarcinoma and squamous cell carcinoma gene expression subtypes demonstrate significant differences in tumor immune landscape. J. Thorac. Oncol..

[25] Huang Y-T (2011). Cigarette smoking increases copy number alterations in nonsmall-cell lung cancer. Proc. Natl. Acad. Sci. U. S. A..

[26] Buro-Auriemma LJ (2013). Cigarette smoking induces small airway epithelial epigenetic changes with corresponding modulation of gene expression. Hum. Mol. Genet..

[27] Yang J (2017). Smoking-dependent distal-to-proximal repatterning of the adult human small airway epithelium. Am. J. Respir. Crit. Care Med..

[28] Travis WD (2015). The 2015 world health organization classification of lung tumors: Impact of genetic, clinical and radiologic advances since the 2004 classification. J. Thorac. Oncol..

[29] Du Y (2017). Lung gene expression analysis (LGEA): An integrative web portal for comprehensive gene expression data analysis in lung development. Thorax.

[30] Du Y, Guo M, Whitsett JA, Xu Y (2015). ‘LungGENS’: A web-based tool for mapping single-cell gene expression in the developing lung. Thorax.

[31] Kim N (2020). Single-cell RNA sequencing demonstrates the molecular and cellular reprogramming of metastatic lung adenocarcinoma. Nat. Commun..

[32] The Gene Ontology Consortium (2017). Expansion of the gene ontology knowledgebase and resources. Nucleic Acids Res..

[33] Fabregat A (2018). The reactome pathway knowledgebase. Nucleic Acids Res..

[34] Liebler JM (2015). Combinations of differentiation markers distinguish subpopulations of alveolar epithelial cells in adult lung. Am. J. Physiol..

[35] Han S, Mallampalli RK (2015). The role of surfactant in lung disease and host defense against pulmonary infections. Ann. Am. Thorac. Soc..

[36] Moré JM (2010). Smoking reduces surfactant protein D and phospholipids in patients with and without chronic obstructive pulmonary disease. BMC Pulm. Med..

[37] Honda Y, Takahashi H, Kuroki Y, Akino T, Abe S (1996). Decreased contents of surfactant proteins A and D in BAL fluids of healthy smokers. Chest.

[38] Sin DD, Man SFP, McWilliams A, Lam S (2008). Surfactant protein D and bronchial dysplasia in smokers at high risk of lung cancer. Chest.

[39] Macía I (2017). P1.05–004 surfactant protein C is a prognostic marker in resected non-small cell lung cancer: Topic: Translational research and biomarkers. J. Thorac. Oncol..

[40] Herzig MC (2011). LRRK2 protein levels are determined by kinase function and are crucial for kidney and lung homeostasis in mice. Hum. Mol. Genet..

[41] Nayak A, Dodagatta-Marri E, Tsolaki AG, Kishore U (2012). An insight into the diverse roles of surfactant proteins, SP-A and SP-D in innate and adaptive immunity. Front. Immunol..

[42] Lavin Y (2017). Innate immune landscape in early lung adenocarcinoma by paired single-cell analyses. Cell.

[43] Aran D, Sirota M, Butte AJ (2015). Systematic pan-cancer analysis of tumour purity. Nat. Commun..

[44] Beauchemin KJ (2016). Temporal dynamics of the developing lung transcriptome in three common inbred strains of laboratory mice reveals multiple stages of postnatal alveolar development. PeerJ.

[45] Eyre TA, Wright MW, Lush MJ, Bruford EA (2007). HCOP: A searchable database of human orthology predictions. Brief. Bioinform..

[46] Wright MW, Eyre TA, Lush MJ, Povey S, Bruford EA (2005). HCOP: The HGNC comparison of orthology predictions search tool. J. Int. Mamm. Genome Soc..

[47] Phipson B, Smyth GK (2010). Permutation p-values should never be zero: calculating exact p-values when permutations are randomly drawn. Stat. Appl. Genet. Mol. Biol..

[48] Tripathi S (2015). Meta- and orthogonal integration of influenza ‘OMICs’ data defines a role for UBR4 in virus budding. Cell Host Microbe.

[49] Amy RW, Bowes D, Burri PH, Haines J, Thurlbeck WM (1977). Postnatal growth of the mouse lung. J. Anat..

[50] Pozarska A (2017). Stereological monitoring of mouse lung alveolarization from the early postnatal period to adulthood. Am. J. Physiol..

[51] Crocker TT, Teeter A, Nielsen B (1970). Postnatal cellular proliferation in mouse and hamster lung. Cancer Res..

[52] Mund SI, Stampanoni M, Schittny JC (2008). Developmental alveolarization of the mouse lung. Dev. Dyn..

[53] Herring MJ, Putney LF, Wyatt G, Finkbeiner WE, Hyde DM (2014). Growth of alveoli during postnatal development in humans based on stereological estimation. Am. J. Physiol. Lung Cell. Mol. Physiol..

[54] Desai TJ, Brownfield DG, Krasnow MA (2014). Alveolar progenitor and stem cells in lung development, renewal and cancer. Nature.

[55] Vogel ER (2015). Perinatal oxygen in the developing lung. Can. J. Physiol. Pharmacol..

[56] Miller YE (2003). Induction of a high incidence of lung tumors in C57BL/6 mice with multiple ethyl carbamate injections. Cancer Lett..

[57] Thorsson V (2018). The immune landscape of cancer. Immunity.

[58] Isella C (2017). Selective analysis of cancer-cell intrinsic transcriptional traits defines novel clinically relevant subtypes of colorectal cancer. Nat. Commun..

[59] Ringnér M, Jönsson G, Staaf J (2016). Prognostic and chemotherapy predictive value of gene-expression phenotypes in primary lung adenocarcinoma. Clin. Cancer Res..

[60] Dächsel JC (2010). A comparative study of Lrrk2 function in primary neuronal cultures. Parkinsonism Relat. Disord..

[61] Nikitin AY (2004). Classification of proliferative pulmonary lesions of the mouse: Recommendations of the mouse models of human cancers consortium. Cancer Res..

[62] Zhang AW (2018). Interfaces of malignant and immunologic clonal dynamics in ovarian cancer. Cell.

[63] Davies P (2013). Comprehensive characterization and optimization of anti-LRRK2 (leucine-rich repeat kinase 2) monoclonal antibodies. Biochem. J..

