# Supplementary material for: Loss of Parkinson’s susceptibility gene LRRK2 promotes carcinogen-induced lung tumorigenesis
Source: Sci Rep. 2021 Jan 22;11:2097. doi: 10.1038/s41598-021-81639-0 (PMC7822882; doi:10.1038/s41598-021-81639-0)
Supplement: Supplementary file 7 — Supplementary Information. [file 41598_2021_81639_MOESM7_ESM.pdf]

Supplementary Materials

Loss of Parkinson's susceptibility gene LRRK2 promotes carcinogen-induced lung tumorigenesis

Chandra Lebovitz, Nicole Wretham, Maryam Osooly, Katy Milne, Tia Dash, Shelby Thornton, Basile Tessier-Cloutier, Paalini Sathiyaseelan, Svetlana Bortnik, Nancy Erro Go, Elizabeth Halvorsen, Rachel Cederberg, Norman Chow, Nancy Dos Santos, Kevin L. Bennewith, Brad H. Nelson, Marcel B. Bally, Wan L. Lam, Sharon M. Gorski

Table of Contents

Supplemental Methods…………………………………………………………………..Page 3

Supplementary Figure S1………………………………………………………………..Page 4

Supplementary Figure S2………………………………………………………………..Page 5

Supplementary Figure S3………………………………………………………………..Page 6

Supplementary Figure S4………………………………………………………………..Page 7

Supplementary Figure S5………………………………………………………………..Page 8

Supplementary Figure S6………………………………………………………………..Page 9

Supplementary Figure S7………………………………………………………………..Page 10

Supplemental References……..……………………………………………………………Page 11

Supplemental Methods

Analysis of single cell RNA-sequencing of human lung

Raw unique molecular identifier (UMI) counts, generated by Kim et al.[1] were downloaded from the Gene Expression Omnibus (accession GSE131907). The following quality measures were applied to each cell's raw gene-cell-barcode matrix: mitochondrial genes ≤20% UMI counts, with UMI count and gene count ranging from 100 to 150,000 and 200 to 10,000, respectively. Raw UMI counts were normalized and transformed, and the most variable genes identified, using the standard workflow for single cell RNA-seq data provided by the Seurat package (version 3.0; R version 3.6.3). Briefly, we applied the SCTransform command with vars.to.regress = "percent.mt". Dimensionality reduction by Uniform Manifold Approximation and Projection for Dimension Reduction (UMAP) and cell clustering was achieved using the following commands, with default parameters and an input of the first 30 principal components identified by PCA in subsequent analyses: RunPCA, RunUMAP, FindNeighbors, FindClusters. Differential expression, between alveolar type II cells and all other cell types grouped, was tested using the FindMarkers command (Wilcoxon rank sum test and Bonferroni multiple comparison correction).

Dimensionality-reduction of TCGA LUAD RNA-seq data using Principal Component Analysis (PCA)

Normalized RSEM counts from both tumor and normal lung samples were downloaded from Level 3 TCGA LUAD RNA-seq data (doi:10.7908/C11G0KM9), filtered for lowly expressing genes (counts per million > 1 in at least the minimum group sample size of interest (*LRRK2*-low); n=14,752 genes or 71.9% of detected genes), and transformed using the varianceStabilizingTransformation command from the DESeq2 package (version 1.28; R version 4.0.2). PCA was performed using the standard workflow of the PCAtools package (version 2.1.2; R version 4.0.2). As top contributors to the most significant principal components (PCs) did not change when inputing either all genes or between the top 5-30% most variant genes, while inputing the most highly variant genes maximized the total variation explained, we chose to include only the top 5% most variant genes as input for our final results (n=737 genes). Applying the findElbowPoint command to the filtered dataset defined the first 14 principal components (PC14) as the most significant, which accounted for 60.1% of the variation among samples. This analysis generated two data matrices: gene loading values (the correlation between each gene and each PC) and sample scores (the relationship between each sample and each principal component) (Table S6).

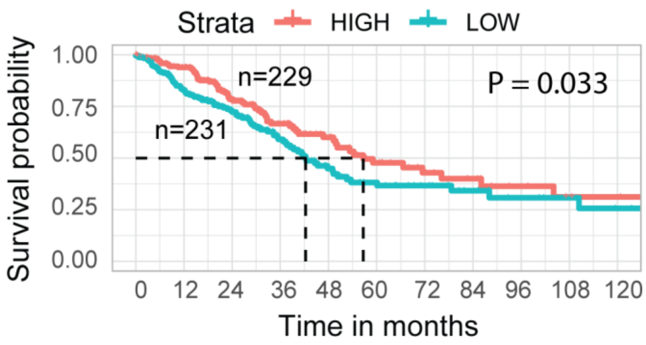

Supplementary Figure S1 (Related to Fig 1A-B). TCGA LUAD overall survival difference, in patients with low versus high *LRRK2* tumoral expression, using median tumoral expression of *LRRK2* as cutpoint.

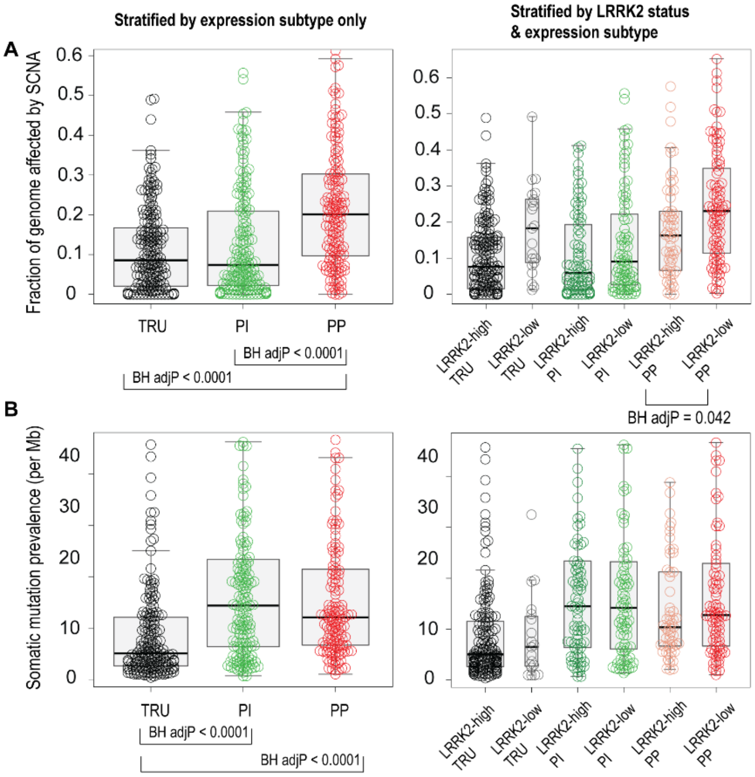

Supplementary Figure S2. ***LRRK2*-low tumors are associated with increased, subtype-specific genome-wide somatic copy number load but not with changes to mutation prevalence.** (A) Plot of somatic copy number load, defined as the count of nucleotides affected by copy number alteration (GISTIC; segmented value > 0.3) normalized to the total number of nucleotides contained in all segments identified per patient, stratified by expression subtype status (Left) or both *LRRK2* and expression subtype status (Right) (n = 472 total tumor samples; median-centred boxplots). Although the test between *LRRK2*-high TRU and -low TRU is significant (BH adjP = 0.0085), we note that there is a drastic sample imbalance that makes inferences from this result problematic. (B) Plot of somatic mutation prevalence per megabase (Mb), defined as the count of all somatic mutations per patient normalized to an approximate protein-coding exome size of 30 Mb, stratified by expression subtype status (Left) or combined LRRK2 and expression subtype status (Right) (n = 475 total tumor samples; median-centred boxplots). BH adjP, Benjamini Hochberg adjusted p-value for Dunn's Test.

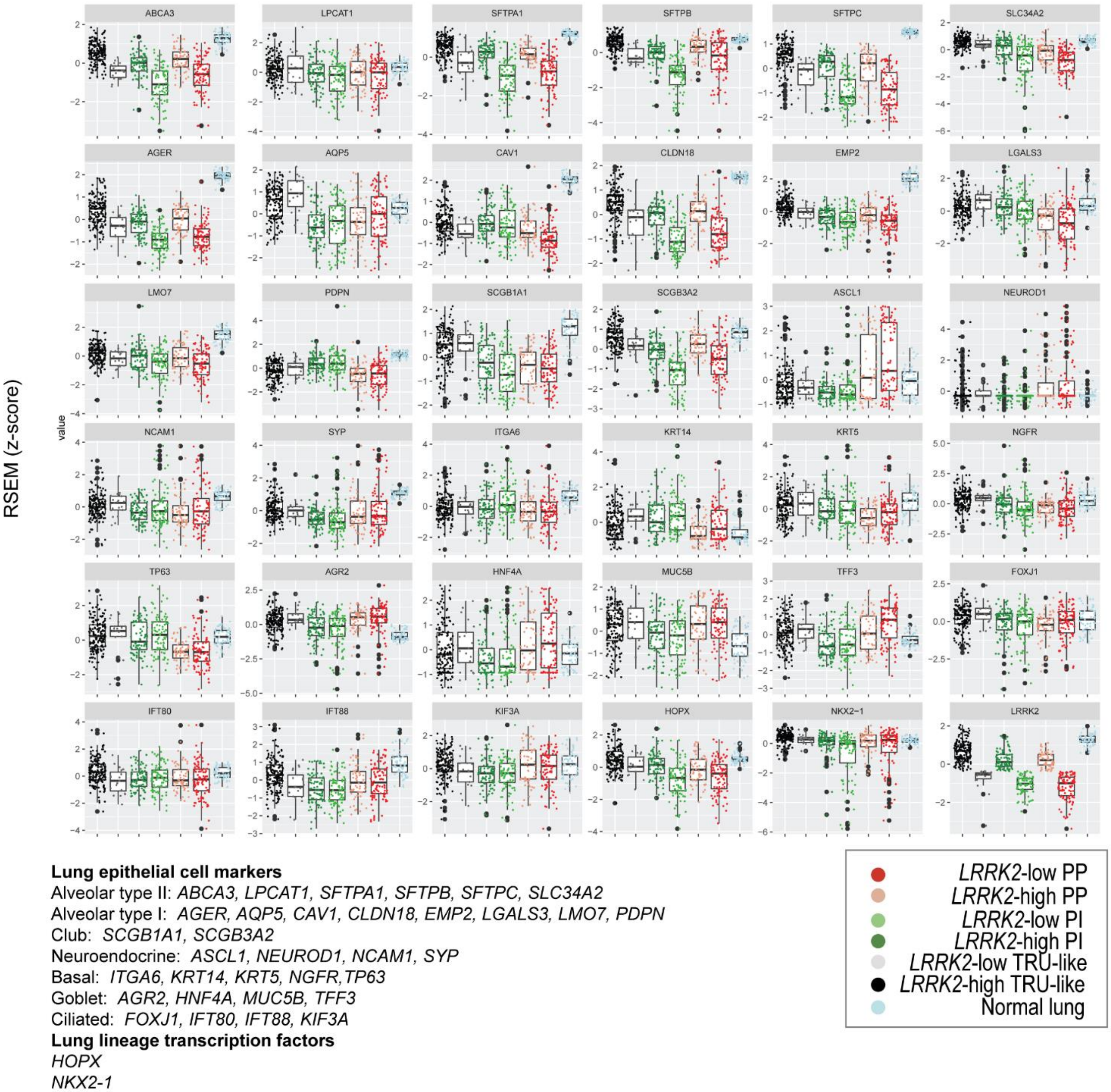

Supplementary Figure S3 (related to Fig 1G). Established lung epithelial cell marker gene expression stratified by the *LRRK2* and expression subtype status of TCGA LUAD tumors. Median centred boxplots of the tumoral expression of lung epithelial cell gene markers, reported by The Lung Gene Expression Analysis Web Portal and curated by the 'LungMAP' consortium, stratified by tumor group.

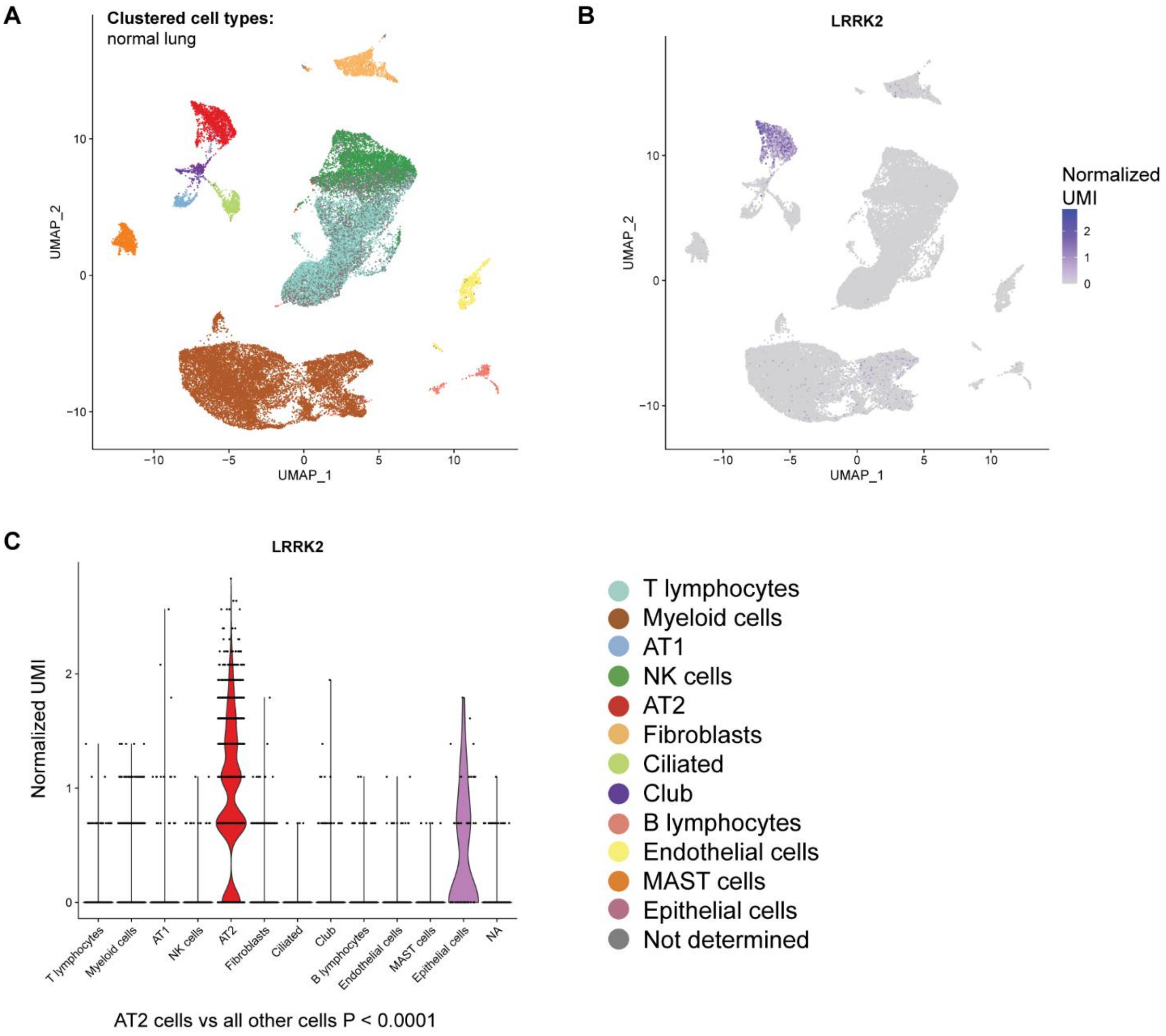

Supplementary Figure S4 (Related to Fig 1G). Cell type specific expression of *LRRK2* in normal human lung.

(A) UMAP visualization of clustered cell types, annotated by Kim et al.[1] in normal human lung.

(B) *LRRK2* expression level, visualized per normal lung cell (Normalized UMI counts).

(C) Violin plot of *LRRK2* expression, across cell types annotated by Kim et al.[1] in normal human lung. Difference in *LRRK2* levels tested between AT2 cells and all other cells grouped (Wilcoxon rank sum test $P < 0.0001$).

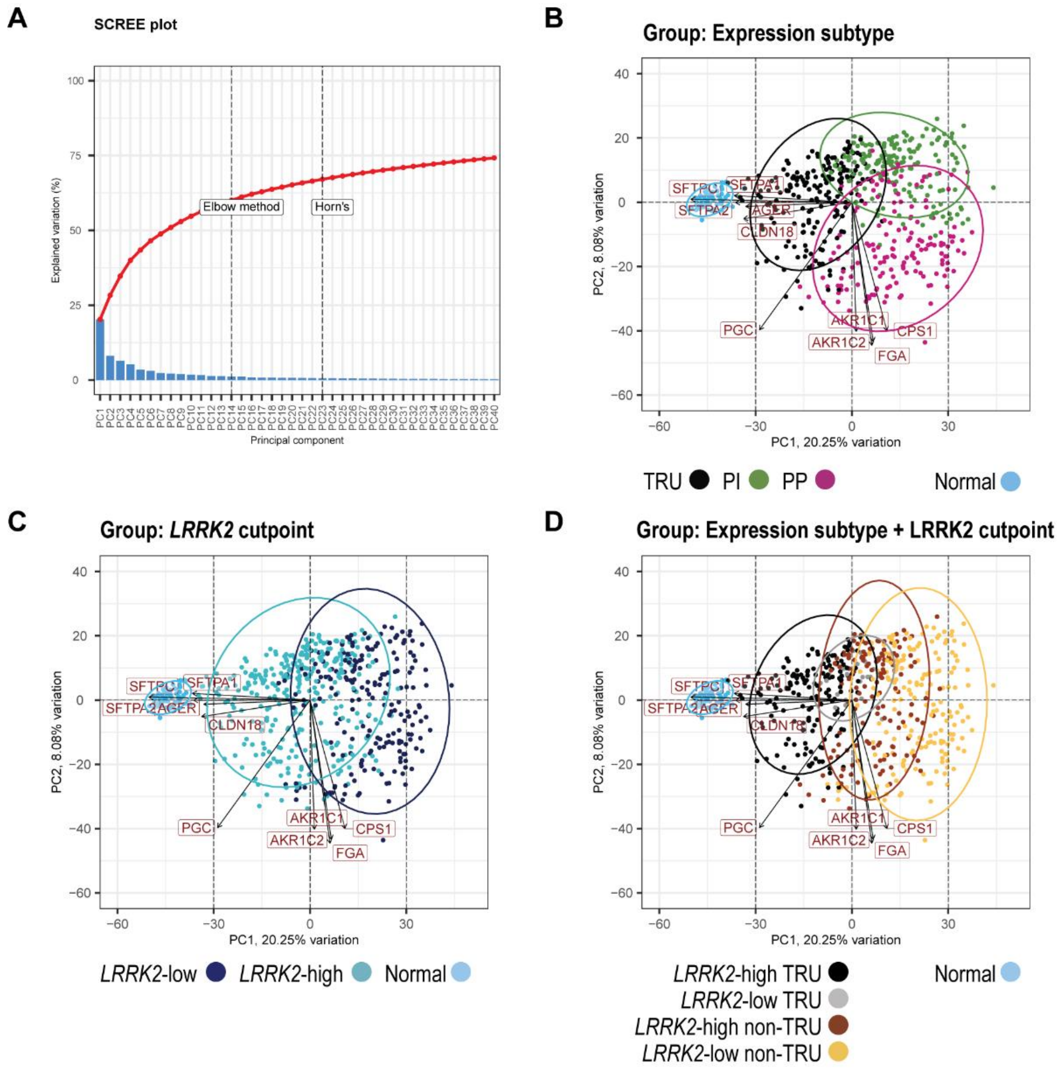

Supplementary Figure S5 (Related to Fig 1G). Dimensionality-reduction of TCGA LUAD RNA-seq data using principal component analysis.

(A) Scree plot summarizing the distribution of explained variance across the top 40 principal components.

(B) Biplot of the first two principal components, annotated with the top 5 gene contributors per component and coloured by the expression subtype status of each sample. TRU: terminal-respiratory unit; PI: proximal-inflammatory; PP: proximal-proliferative.

(C) Biplot of the first two principal components, annotated with the top 5 gene contributors per component and coloured by the *LRRK2* expression status of each sample.

(D) Biplot of the first two principal components, annotated with the top 5 gene contributors per component and coloured by the combined expression subtype and *LRRK2* expression status of each sample. For simplicity, the PI and PP subtypes have been combined into their recognized overarching subtype, referred to in the literature as non-terminal respiratory unit (non-TRU).

*Stat ellipses are drawn around each group at the 95% confidence level.

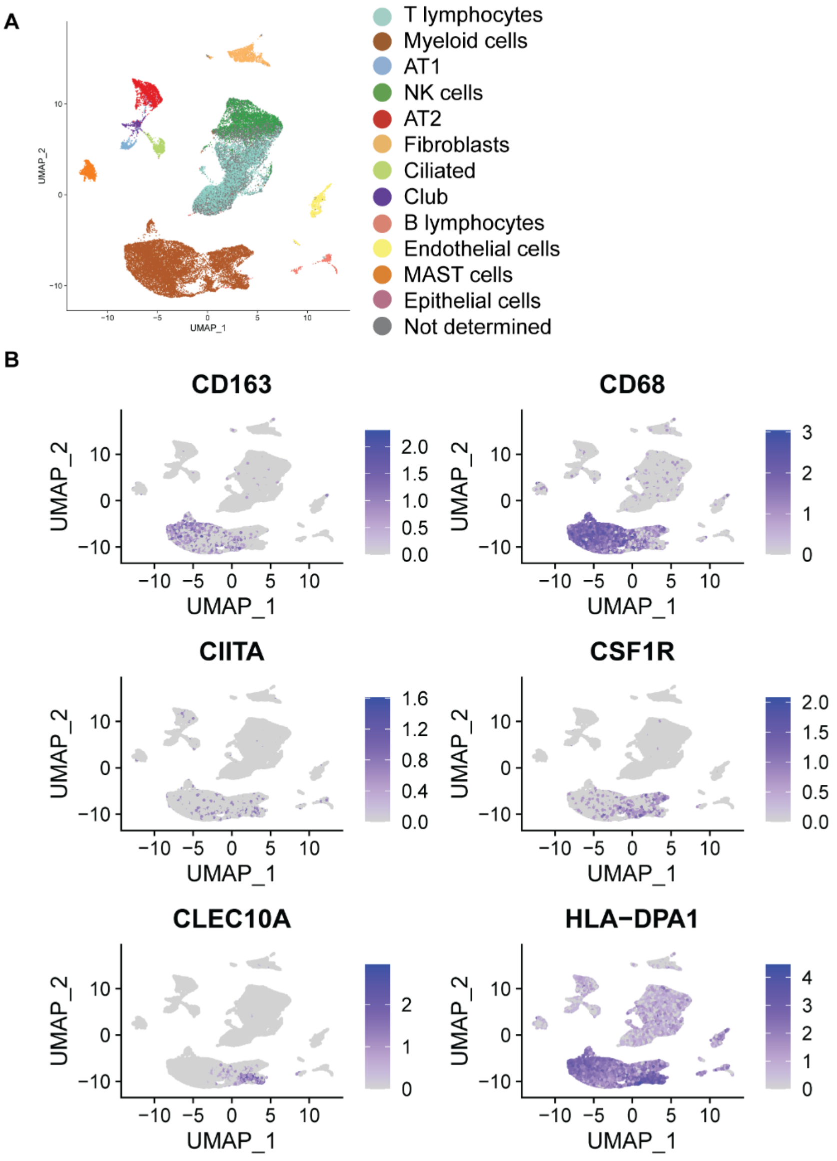

	Avg Log FC	%Myeolid cells	%Other cells	Wilcoxon rank sum test
CD163	0.443	36.7	1.20	P < 0.0001
CD68	1.52	88.8	9.50	P < 0.0001
CIITA	n.d.	n.d.	n.d.	n.d.
CSF1R	0.261	24.1	0.60	P < 0.0001
CLEC10A	0.267	11.4	0.30	P < 0.0001
HLA-DPA1	1.52	98.5	64.6	P < 0.0001

Supplementary Figure S6 (Related to Fig 2C). Cell type specific expression of various myeloid cell markers and MHC-II gene *HLA-DPA1* in normal human lung.

(A) UMAP visualization of clustered cell types, annotated by Kim et al.[1] in normal human lung.

(B) Expression levels of various myeloid cell markers and MHC-II pathway genes visualized per normal lung cell (normalized UMI counts), with summarized statistics of differential expression between myeloid cells and all other cells.

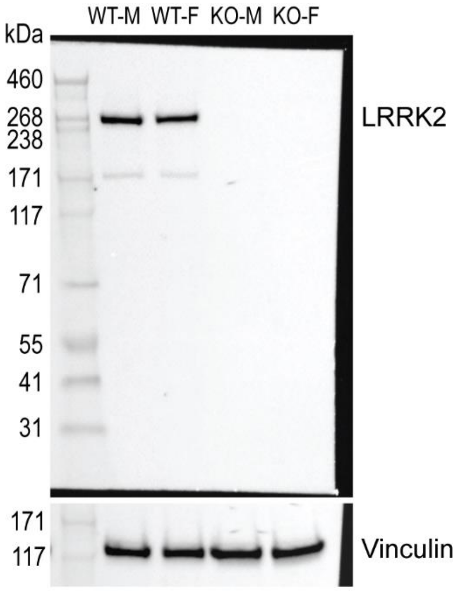

Supplementary Figure S7 (Related to Fig 3D-F). Validation of LRRK2 antibody via western blot analysis of mouse lung tissue lysates.

The LRRK2 antibody (clone N241A/34, 75-253, Antibodies Incorporated) was also used for IHC analyses on FFPE mouse lung in Figure 3.

WT = homozygous LRRK2 wildtype (derived from heterozygous C57BL/6-Lrrk2^{tm1Mjfa} line);
KO = homozygous LRRK2 knockout (derived from heterozygous C57BL/6-Lrrk2^{tm1Mjfa} line);

-M = male; -F = female

Supplemental References

1. Kim, N. *et al.* Single-cell RNA sequencing demonstrates the molecular and cellular reprogramming of metastatic lung adenocarcinoma. *Nat. Commun.* **11**, 2285 (2020).
